# A Prospective Feasibility Trial to Challenge Patient–Derived Pancreatic Cancer Organoids in Predicting Treatment Response

**DOI:** 10.3390/cancers13112539

**Published:** 2021-05-21

**Authors:** Alica K. Beutel, Lena Schütte, Jeanette Scheible, Elodie Roger, Martin Müller, Lukas Perkhofer, Annika M. T. U. Kestler, Johann M. Kraus, Hans A. Kestler, Thomas F. E. Barth, Johannes Lemke, Marko Kornmann, Thomas J. Ettrich, Johann Gout, Thomas Seufferlein, Alexander Kleger

**Affiliations:** 1 Department of Internal Medicine, University Hospital Ulm, 89081 Ulm, Germany; alica.beutel@uniklinik-ulm.de (A.K.B.); lena.schuette@uniklinik-ulm.de (L.S.); jeanette.scheible@uni-ulm.de (J.S.); elodie.roger@uni-ulm.de (E.R.); martin.mueller@uniklinik-ulm.de (M.M.); lukas.perkhofer@uniklinik-ulm.de (L.P.); thomas.ettrich@uniklinik-ulm.de (T.J.E.); johann.gout@uni-ulm.de (J.G.); 2 Institute of Medical Systems Biology, Ulm University, 89081 Ulm, Germany; annika.kestler@uni-ulm.de (A.M.T.U.K.); johann.kraus@uni-ulm.de (J.M.K.); hans.kestler@uni-ulm.de (H.A.K.); 3 Institute of Pathology, University Hospital Ulm, 89081 Ulm, Germany; thomas.barth@uniklinik-ulm.de; 4 Department of General and Visceral Surgery, University Hospital Ulm, 89081 Ulm, Germany; johannes.lemke@uniklinik-ulm.de (J.L.); marko.kornmann@uniklinik-ulm.de (M.K.)

**Keywords:** pancreatic cancer, organoids, drug response prediction, pharmacotyping, personalized medicine

## Abstract

**Simple Summary:**

Pancreatic cancer (PC) is characterized by an exceptionally aggressive tumor biology, high inter- and intratumor heterogeneity, and resistance to conventional chemotherapy, targeted agents, and immunotherapy. With its rising incidence and dismal prognosis, PC is projected to become the second-leading cause of cancer-related death worldwide in 2030. Tumor heterogeneity induces a considerable variation in responses to antitumor therapies, yet reliable models or biomarkers to predict the effectiveness of treatment strategies for eligible subgroups are not established. Current combination chemotherapeutic regimens are often ineffective and frequently exhibit substantial systemic toxicity impeding longer-term treatment. Patient-derived pancreatic cancer organoids (PDOs) exhibit features of the parental tumor and may thereby represent a powerful preclinical tool to predict drug response. Ex vivo pharmacotyping may enable therapy response prediction and harness personalized treatment in PC patients. In clinical practice, a PDO-guided selection of effective drugs may provide substantial benefit and improve survival outcomes in this heterogeneous disease.

**Abstract:**

Real-time isolation, propagation, and pharmacotyping of patient-derived pancreatic cancer organoids (PDOs) may enable treatment response prediction and personalization of pancreatic cancer (PC) therapy. In our methodology, PDOs are isolated from 54 patients with suspected or confirmed PC in the framework of a prospective feasibility trial. The drug response of single agents is determined by a viability assay. Areas under the curves (AUC) are clustered for each drug, and a prediction score is developed for combined regimens. Pharmacotyping profiles are obtained from 28 PDOs (efficacy 63.6%) after a median of 53 days (range 21–126 days). PDOs exhibit heterogeneous responses to the standard-of-care drugs, and are classified into high, intermediate, or low responder categories. Our developed prediction model allows a successful response prediction in treatment-naïve patients with an accuracy of 91.1% for first-line and 80.0% for second-line regimens, respectively. The power of prediction declines in pretreated patients (accuracy 40.0%), particularly with more than one prior line of chemotherapy. Progression-free survival (PFS) is significantly longer in previously treatment-naïve patients receiving a predicted tumor sensitive compared to a predicted tumor resistant regimen (mPFS 141 vs. 46 days; *p* = 0.0048). In conclusion, generation and pharmacotyping of PDOs is feasible in clinical routine and may provide substantial benefit.

## 1. Introduction

Pancreatic cancer (PC) still has a dismal prognosis with a 5-year survival rate of only 8% in all stages combined [1]. The main reasons are late diagnosis due to lack of specific early symptoms, exceptionally aggressive tumor biology with substantial inter- and intratumor heterogeneity, and resistance to chemotherapy, targeted agents, and immunotherapy. The majority of patients (>80%) are diagnosed at an advanced tumor stage that renders them ineligible for surgical resection, currently the only potentially curative treatment, at best combined with (neo)adjuvant chemotherapy. In contrast to other solid tumors, little progress has been made using targeted treatments based on individual genomic features in PC. Less than 2% of PC patients referred for molecular profiling ultimately received a matched therapy [2]. Thus, conventional chemotherapy still represents the mainstay in the treatment of PC patients. The current standard-of-care first-line regimen in the advanced setting is either gemcitabine/nab-paclitaxel [3] or FOLFIRINOX (5-fluorouracil, leucovorin, irinotecan, and oxaliplatin) [4], usually guided by patients’ performance status and age. After these combinations, up to 50% of patients received second-line chemotherapy [5,6]. 5-fluorouracil plus either (nanoliposomal) irinotecan or oxaliplatin is frequently applied upon progress under gemcitabine/nab-paclitaxel [7,8]. For patients with prior FOLFIRINOX therapy, gemcitabine alone or in combination with nab-paclitaxel may be offered, however, with limited clinical evidence [9]. Albeit more than 20% of patients receive three or more lines of therapy, there is no clear recommendation based on clinical trials [5,10]. Driver mutations in pancreatic cancer, such as *KRAS*, *TP53*, *CDKN2A*, *SMAD4*, and several more, are mostly not druggable, but generate an exceptional inter- and intratumor heterogeneity [11,12], inducing a considerable variation in treatment response. Current combinational cytotoxic regimens exhibit considerable side effects, frequently requiring dose reductions and hospitalization, which severely impacts quality of life and often impedes longer-term treatment. Moreover, potentially effective drugs for certain PC subgroups frequently fail to demonstrate clinical activity, due to the lack of reliable predictive models or biomarkers in clinical routine identifying eligible patients. Such models to select effective therapies for subgroups of patients and to prevent toxicity from ineffective drugs are urgently warranted, particularly for a disease as heterogeneous as PC.

Patient-derived xenograft (PDX) and patient-derived organoid (PDO) models have proven as powerful tools in basic and translational research. PDX models preserve biological features of the parental tumor and the complex interplay with the tumor microenvironment [13,14]. They also proved to correlate with the drug prediction of PDX tumor-generated organoid (PXO) models [15] and reproduced therapeutic responses observed in patients with gastrointestinal malignancies [14,16,17]. However, the time-consuming and labor-intensive generation of PDX decisively limits their usefulness for a timely preclinical screening of multiple drugs. In contrast, PDOs represent a suitable method for ex vivo pharmacotyping within a reasonable time frame [18]. PDO-derived gene signatures in PC were found to predict chemotherapy sensitivity [19], and in vitro drug responses correlated with clinical response in various patient examples in gastrointestinal cancers [20,21]. However, a prospective assessment of the pharmacotyping profiles with the therapeutic responses and clinical outcome in a larger number of PC patients is, to our knowledge, not yet available. Thus, its potential for informing clinical decision-making remains elusive.

In this prospective trial, we evaluated the feasibility of PDOs as a preclinical tool for drug response prediction in a clinical routine setting and correlated the in vitro prediction with the therapeutic response in a representative cohort of PC patients.

## 2. Materials and Methods

### 2.1. Patient Selection and Ethics Statement

Patients with a presumed or confirmed diagnosis of PC, regardless of tumor stage or treatment status, were eligible to participate in the prospective research project at the University Hospital Ulm. Patients with PC suspicion underwent routine diagnostic work-up and tissue acquisition to confirm the diagnosis or surgical resection independently of trial participation. Pretreated patients who were re-biopsied for participation in a clinical study or for panel-sequencing, were also included in this prospective trial. Exclusion criteria were inability to provide written informed consent or lack of clinical indication for invasive tissue acquisition. Patient enrollment in the study was conducted between July 2019 and January 2021 at the University Hospital Ulm. The project was approved by the local ethics committee (project number 72/19), and written informed consent was obtained from all patients.

### 2.2. Tumor Specimen Collection

All ultrasound (US)-guided procedures were performed at the Department of Internal Medicine 1 of the University Hospital Ulm. One or two tumor specimens (sampled with a 16- or 18-gauge needle for US-guided biopsies of the primary tumor and liver metastases; a standard 19-gauge needle for endoscopic ultrasound (EUS)-guided biopsies) were processed for organoid isolation. Surgical specimens were received intraoperatively from the Department of General and Visceral Surgery of the University Hospital Ulm. Samples were placed into advanced DMEM/F12 medium supplemented with 100 µg/mL primocin and 10 mg/mL bovine serum albumin, and were kept on ice until isolation (within 30 min to 3 h). Diagnosis of PC was confirmed by routine pathology assessment.

### 2.3. Isolation and Culture of Patient-Derived Organoids

Briefly, tissue biopsy samples and surgical tumor pieces were minced into 1 mm fragments with a sterile scalpel and enzymatically digested with accutase (Sigma-Aldrich, Merck, Darmstadt, Germany) for 40 min at 37 °C. Cells were then seeded into Matrigel Growth Factor Reduced (GFR) (Corning, New York, NY, USA) domes covered with organoid culture medium containing WNT3A/RSPOI-conditioned medium [22]. Organoids were propagated at 37 °C under 5% (*v*/*v*) CO_2_ atmosphere and passaged for cell line expansion or characterization every 5 to 10 days depending on cell density and growth rate. Mycoplasma tests were regularly performed using the MycoSPX PCR kit (Biontex, Munich, Germany). WNT signaling pathway activity of the WNT3A-conditioned medium was regularly monitored with a dual luciferase (Firefly-Renilla, BPS Bioscience, Hamburg, Germany) assay system (TCF/LEF reporter kit, BPS Bioscience, Hamburg, Germany).

### 2.4. Pharmacotyping

Prior to seeding, organoids were dissociated into single cells using accutase. Cells were then seeded on a Matrigel GFR-coated 96-well plate (2000 cells per well) in an organoid culture medium containing WNT3A/RSPOI-conditioned medium [22] and 5% Matrigel GFR, one to four days before treatment. After four days of treatment, cell viability was analyzed with the CytoTox-Glo cytotoxicity assay (Promega, Walldorf, Germany) according to the manufacturer’s protocol. Luminescence was measured using a Tecan Infinite M200 Pro (Tecan, Crailsheim, Germany). Chemotherapeutics were tested in duplicates in ten concentrations covering three orders of magnitudes (13 nM to 50 µM). Percentage of cell viability was determined using this formula: (S2−S1)_Drug_/(S2−S1)_Ctrl_. The area under the curve (AUC) was estimated using the trapezoidal rule with GraphPad Prism v8 (GraphPad Software, San Diego, CA, USA). For each single chemotherapeutic substance, AUCs from the PDO library (n ≥ 25) were classified into three subgroups (low, intermediate, and high responder) using the Jenks Natural Breaks classification method. 5-fluorouracil (NSC 19893), docetaxel (NSC 628503), doxorubicin (adriamycin), etoposide, everolimus (RAD001), gemcitabine (LY188011), irinotecan (CPT-11), lenvatinib (E7080), mitomycin C, oxaliplatin (L-OHP), and paclitaxel (NSC 125973) were purchased from Selleckchem. Of note, irinotecan, instead of its metabolite SN-38, has been employed as it has been shown to exert antitumor activity in vitro [23,24,25] even if it is less cytotoxic than SN-38 [24].

### 2.5. Immunostaining

PDOs were fixed in a solution of 4% phosphate-buffered paraformaldehyde and 10% sucrose overnight at 4 °C and embedded in paraffin. Immunohistochemical analyses were performed on serial sections of 4 μm using standard techniques. The primary antibodies used were: mouse monoclonal antibodies against Ki-67 (1:200; clone MIB-1, Dako, Agilent, Santa Clara, CA, USA) and rabbit monoclonal antibodies against GATA6 (1:100; clone D61E4, Cell Signaling Technology, Danvers, MA, USA). GATA6 and Ki-67 scorings were performed by a board-certified pathologist blinded to patients’ clinical information. Clustering into highly proliferative (Ki-67 positivity > 45%) and low proliferative (Ki-67 positivity ≤ 45%) subgroups were determined by the Jenks Natural Breaks classification method. Bright-field images were obtained using an HCX PL APO 40×/0.85 (Leica, Wetzlar, Germany) objective mounted on a Leica DM5500 B microscope equipped with a Leica DFC420C camera and Leica Application Suite software (Leica, Wetzlar, Germany).

### 2.6. Patients’ Evaluation of Therapeutic Response

Treatment response was routinely evaluated by computed tomography (CT) scan every 8–12 weeks during therapy. Analyses were based on RECIST 1.1 (Response Evaluation Criteria In Solid Tumors) as a standardized measurement for tumor response.

### 2.7. Linear Support Vector Machines

Two linear support vector machines (SVMs) were trained on AUCs of 11 untreated PDO samples, labeled “response” and “no response” according to the patients’ treatment outcome. The first SVM was trained on patients who received FOLFIRINOX treatment, and the second SVM was trained on patients who received gem/nab-p treatment. Both SVM resulted in a perfectly separating hyperplane with three support vectors each (FOLFIRINOX “response”: PDO 5, PDO 14; “no response”: PDO 3; gem/nab-p “response”: PDO 17; “no response”: PDO 28, PDO 54). The performance of the classifier was tested on an independent sample (PDO 07). Both SVMs correctly predicted treatment outcomes in this patient for first and second-line therapy.

### 2.8. Statistical Analysis

GraphPad Prism v8 (GraphPad Software, San Diego, CA, USA) was used for statistical analysis. Kaplan-Meier analysis was used for the calculation of progression-free survival (PFS). Statistical significance in contingency tables was tested using Fisher’s exact test. All statistical tests were considered to be statistically significant when *p* < 0.05.

## 3. Results

### 3.1. Prospective Feasibility Trial

**Figure 1:**
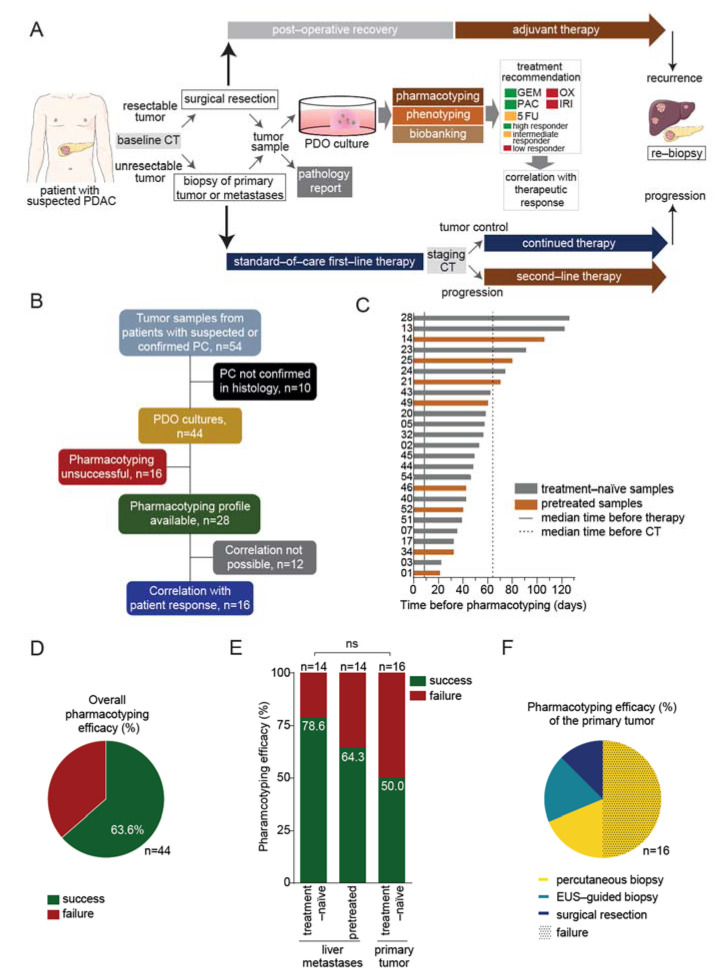
Feasibility of organoid-based precision medicine in clinical routine. (**A**), Schematic representation of the work-flow. Patient-derived organoids (PDOs) were isolated from surgical specimens or biopsy samples of the primary tumor or liver metastases in pancreatic cancer (PC) patients. PDOs were submitted to pharmacotyping, phenotyping, and biobanking. The in vitro prediction was compared to the therapeutic response in the patient. (**B**), Schematic representation of the study. (**C**), Graphic representation showing the time required before PDO pharmacotyping. (**D**), Representation of the overall PDO pharmacotyping efficacy. (**E**), Representation of the pharmacotyping efficacy in PDOs isolated from liver metastases of either treatment-naïve or pretreated patients, or from the primary tumor. (**F**), Pie chart showing the pharmacotyping efficacy of the primary tumor according to the sampling method (percutaneous biopsy, *n* = 11; endoscopic ultrasound (EUS)-guided biopsy, *n* = 3; surgical resection, *n* = 2); dotted pattern depicts pharmacotyping failure. 5-FU, 5-fluorouracil; GEM, gemcitabine; IRI, irinotecan; OX, oxaliplatin; PAC, paclitaxel; CT, computed tomography; ns, not significant.

Patients with a presumed or confirmed diagnosis of PC were prospectively included in a feasibility trial at the University Hospital Ulm, regardless of tumor stage or treatment status. Isolation of organoids was performed using surgical or biopsy specimens obtained from the primary pancreatic tumor or liver metastasis, respectively (Figure 1A). Patients with a primary diagnosis of histologically confirmed PC received standard-of-care first-line therapy or adjuvant therapy after surgical resection at the discretion of the attending gastrointestinal oncologist based on tumor stage, age, performance status, and comorbidities. Pretreated patients with metastatic disease who underwent biopsy for participation in a clinical study or for panel-sequencing, were also included in this prospective trial. Each PDO underwent a small-scale drug testing screen with the five most commonly used standard-of-care chemotherapy compounds (gemcitabine, paclitaxel, irinotecan, 5-fluorouracil, and oxaliplatin) to obtain a personalized treatment recommendation for a subsequent line of therapy as early as possible (Figure 1A). Individual pharmacotyping profiles were compared afterward to the treatment response as assessed by imaging in each patient.

In total, organoids were propagated from 54 samples of patients with suspected or confirmed PC (Figure 1B). A routine pathology report confirmed exocrine pancreatic malignancy in 44 samples. The major histological subtype was ductal adenocarcinoma (*n* = 41) alongside three rare PC subtypes (undifferentiated anaplastic carcinoma, squamous cell carcinoma, and acinar cell carcinoma, Table S1). Ten samples were excluded as histopathological diagnosis revealed pancreatic neuroendocrine neoplasms (*n* = 4), lymphoma (*n* = 1), serous cystadenoma (*n* = 1), or non-malignant pancreatic necrosis (*n* = 4) (Table S1). Thirty patients with confirmed PC were included in this trial at the time of the primary diagnosis (treatment-naïve) and 14 patients at the time of tumor progression (pretreated) (Table 1). The majority of the treatment-naïve patients was diagnosed at an advanced tumor stage (metastasized, *n* = 18; locally advanced, *n* = 8; resectable, *n* = 4). Biopsy samples were derived from the primary tumor (*n* = 16) or liver metastases (*n* = 14), by ultrasound (US)-guided biopsy (*n* = 25), endoscopic ultrasound (EUS)-guided fine-needle biopsy (FNB) (*n* = 3), or surgical resection (*n* = 2). All tumor samples from pretreated patients were acquired by US-guided biopsies of liver metastases (Table 1).

### 3.2. Pharmacotyping of Patient-Derived Organoids Is Feasible in a Reasonable Time Frame

Diagnosis at an advanced tumor stage limits therapeutic options and opens only a narrow therapeutic window. Our group previously showed that drug response (pharmacotyping) can be accurately predicted based on PDX-derived PDOs when challenged with a conventional PDX assay [9,12]. Here, we implemented these findings in a prospective setting. Pharmacotyping profiles were successfully obtained in samples from 28 patients with confirmed PC (19 treatment-naïve, 9 pretreated; efficacy 63.6%), while 16 PDO cultures could not be pharmacotyped, due to a lack of emergence of organoid structures and/or failure to continue the growth of organoids beyond passage three (Figure 1B,D). Pharmacotyping efficacy was highest in treatment-naïve PDO lines isolated from liver metastases with 78.6% (11 out of 14 PDO cultures). Pretreatment of the patients showed a tendency towards a decreased PDO pharmacotyping efficacy to 64.3% (9 out of 14; *p* = 0.6776). In pretreated patients (*n* = 14), the last chemotherapy administration took place a median of 21.5 days (range 6–259 days; median of 21.0 days in the successful pharmacotyping group (*n* = 9) vs. 37.0 days in the unsuccessful group (*n* = 5), *p* = 0.8905) prior to tissue collection. The lowest pharmacotyping efficacy with 50%, by trend, was obtained in treatment-naïve primary tumors (8 out of 16; *p* = 0.1424) (Figure 1E). Pharmacotyping was possible in all primary tumor samples obtained using EUS-guided biopsy (*n* = 3) and surgical specimen (*n* = 2). Eight out of 11 samples from percutaneous biopsies of the primary tumor failed (Figure 1F). Pharmacotyping results were obtained after a median time of 53 days (range 21–126 days) and were mostly available (67%, 10/15) in advanced PC patients before the first restaging CT scan, which was performed after a median of 56 days (range 46–86 days) following initiation of systemic treatment (Figure 1C). In patients with an exceptionally aggressive clinical course of disease (*n* = 8 patients with rapid deterioration of the performance status preventing any treatment or restaging), we observed a trend towards an accelerated growth rate of organoids (median time before pharmacotyping 44 days vs. 56 days, *p* = 0.1288).

### 3.3. Drug Response Prediction in PDO Cultures

**Figure 2:**
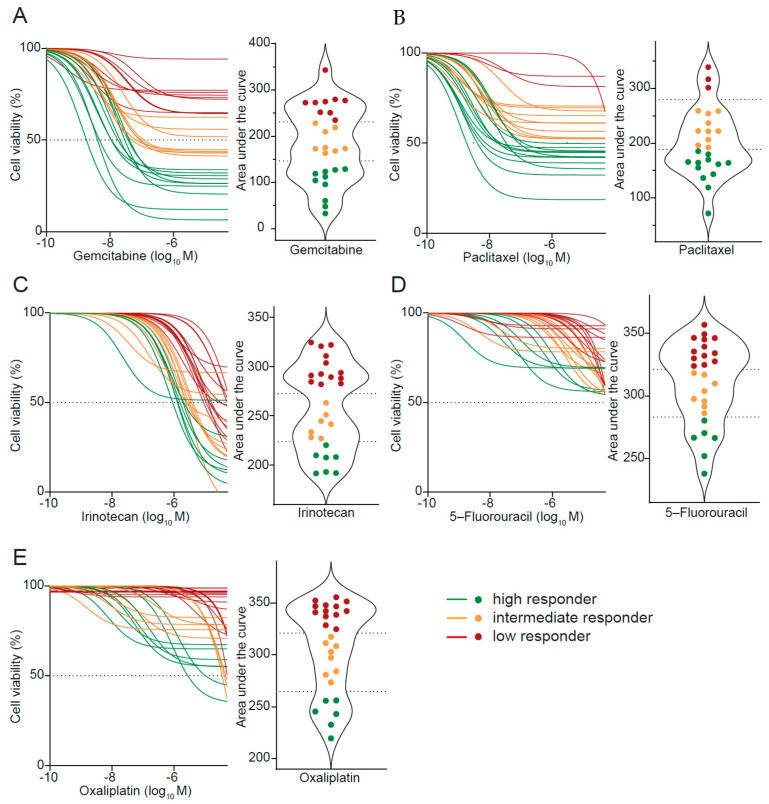
Patient-derived organoids exhibit heterogeneous responses to standard-of-care agents. (**A**–**E**) Cell viability analyses (left panels) and violin plots (right panels) depict corresponding areas under curves of gemcitabine (**A**), paclitaxel (**B**), irinotecan (**C**), 5-fluorouracil (**D**), and oxaliplatin (**E**) treatment in patient-derived organoids (PDOs). Dotted lines (right panels) represent cut-off values as determined by the Jenks Natural Breaks classification method. PDOs were classified into three subgroups: high responder (green), intermediate responder (orange), and low responder (red).

PDO drug sensitivity was assessed by calculating the area under the curve (AUC) upon testing single chemotherapeutic agents. Previously, we showed that the efficiency of a combinatorial regimen is driven by the most potent agent [15]. Thus, testing single compounds offers the advantage to possibly combine potent drugs in novel combinations, while avoiding ineffective substances and thereby toxicity. PDOs exhibited heterogeneous responses to the tested chemotherapies (Figure 2A–E and Figure S4B–G), but robustly operated across various experiments and experimenters (Figure S1A–D). Next, we employed the previously reported Jenks Natural Breaks classification method to prospectively cluster and rank each PDO line—this classifier reduced variance best within a group, while maximizing variance between groups [13,15]. According to the cut-off value for each drug determined by this method, PDO lines could be classified into three subgroups: lowest AUC was defined as high responder, and highest AUC was termed low responder, referring to a negative prediction in terms of efficacy. The third group comprised the intermediate responder group suggesting a limited efficacy in vivo (Figure 2A–E). Best PDO response discriminations were obtained with gemcitabine (high sensitivity AUC < 146.1 and low sensitivity > 231.3) and paclitaxel (high sensitivity < 188.7 and low sensitivity > 280.3) (Figure 2A,B). In contrast, irinotecan, 5-fluorouracil (5-FU), and oxaliplatin induced more homogenous responses across the tested dosages (Figure 2C–E). 5-FU and oxaliplatin were the least effective substances among the five tested (no viability reduction above 50% for 5-FU), while irinotecan induced cytotoxicity at higher dosages, in line with previous literature [19] (Figure 2C–E).

### 3.4. PDO-Based Pharmacotyping Predicts Drug Response to Aid Decision-Making in Patients

**Figure 3:**
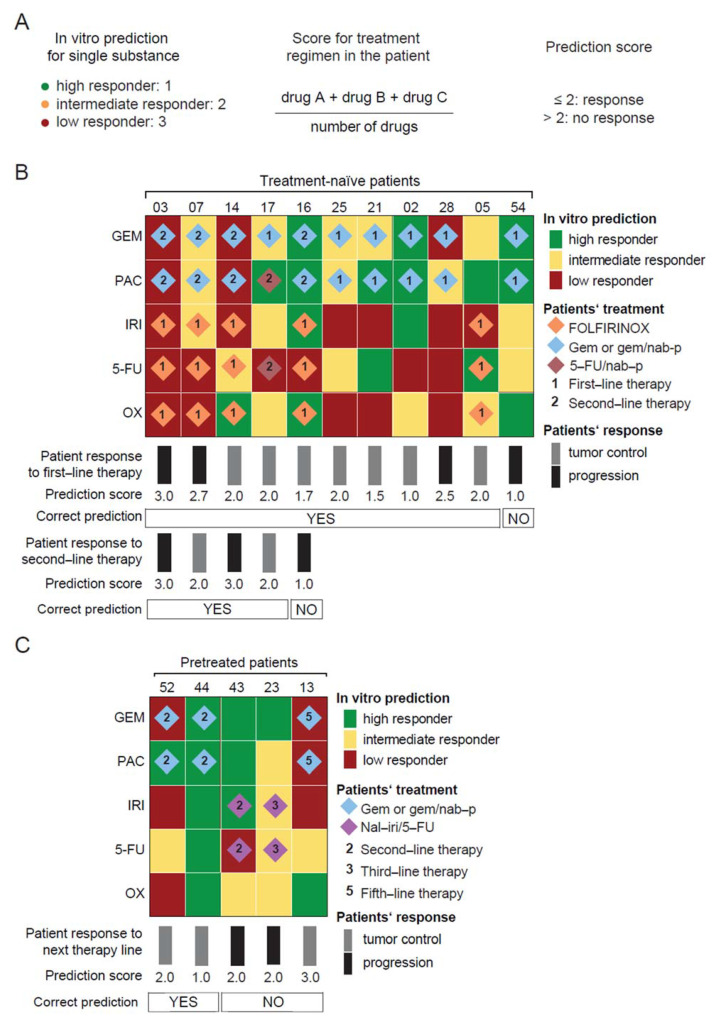
Organoid-based drug efficacy prediction profiles parallel patient therapeutic response. (**A**), Scoring method to predict the patient therapeutic response of a given combinational regimen based on the in vitro prediction for single substances. PDO-based drug response prediction in (**B**), treatment-naïve and (**C**), pretreated PDO samples according to the prediction score shown in (**A**). For each single chemotherapeutic substance, the clustering of the PDO library into high, intermediate, and low responder subgroups was performed using the Jenks Natural Breaks classification method, as shown in Figure 2. Patients’ treatment regimen, therapy line, and therapeutic response as indicated. 5-FU, 5-fluorouracil; FOLFIRINOX, 5-fluorouracil, leucovorin, irinotecan, and oxaliplatin; GEM, gemcitabine; IRI, irinotecan; nab-p, nanoparticle albumin-bound paclitaxel; nal-iri/5-FU, nanoliposomal irinotecan, 5-fluorouracil, and leucovorin; OX, oxaliplatin; PAC, paclitaxel.

A sole drug can increase therapy efficacy within a combinational chemotherapeutic regimen [15]. Therefore, we assigned a score to the AUC subgroup of each drug (high responder = 1, intermediate responder = 2, and low responder = 3) (Figure 2A–E), added up, and divided the sum by the number of drugs included within the regimen to develop a prediction score (Figure 3A). A prediction score below or equal to 2 indicates an efficacious combinational chemotherapy, while a score greater than 2 denotes an inefficacious combination. To validate the prediction score, we correlated clinical response data from 16 out of 28 pharmacotyped patient samples with our PDO–based prediction model. Comparison with clinical outcome was not possible in the remaining 12 patients as they did not receive any chemotherapy (*n* = 5) or restaging despite treatment (*n* = 3), due to rapid deterioration of their performance status, decease from postoperative complications (*n* = 2) or because they were treated with substances not included in our small-scale drug screen (*n* = 2) (Figure 1B).

The in vitro prediction was compared with the therapeutic response in 11 previously treatment-naïve patients (Figure 3B) and in five pretreated patients (Figure 3C). Clinical response was routinely monitored with CT scan during treatment and analyzed based on RECIST 1.1 criteria (complete (CR) or partial response (PR), and stable disease (SD) were designated as tumor control) (Figure S2A–C). In the untreated cohort, seven patients achieved a tumor control, whereas four patients progressed under first-line chemotherapy with FOLFIRINOX (*n* = 5) or gemcitabine/(nab-paclitaxel) (*n* = 6) at the time of the first restaging (Figure 3C). Overall, our PDO-based prediction model correctly predicted therapeutic response to the first-line regimen in 10 out of 11 treatment-naïve patients with an accuracy of 91.1%. Interestingly, the prediction only failed in one patient with an undifferentiated anaplastic tumor, a rare and extremely aggressive histological variant of PC [20] (Table S1). Five out of these 11 patients clinically qualified for second-line treatment upon progression under first-line treatment. Again, gemcitabine/nab-paclitaxel (*n* = 4) or 5-FU/nab-paclitaxel (*n* = 1) treatment resulted either in tumor control (*n* = 2) or progression (*n* = 3). To probe whether PDOs could still aid decision-making at this stage, we compared initial PDO predictions with this clinical response. Four out of the five patients’ sensitivity to drugs used in second-line therapy were accurately predicted by our in vitro model relating to an accuracy of 80.0% (Figure 3B). This demonstrates that treatment-naïve organoids still harbor predictive value to guide second-line regimens.

We also established PDOs from biopsies of progressive lesions in five pretreated patients. Subsequent therapies in these patients were gemcitabine/nab-paclitaxel (*n* = 3) or nal-iri/5-FU (*n* = 2). When correlating clinical response with PDO prediction, only 2 out of 5 patients showed matching outcomes resulting in a substantially lower accuracy of 40.0% compared to PDOs derived from treatment-naïve tumors (Figure 3C). Importantly, two out of three patients whose prediction was correct had previously received only one line of chemotherapy as compared to the heavily pretreated patients whose prediction failed (with 1, 2, and 4 prior lines of therapy, respectively).

### 3.5. A Machine Learning Classifier as an Alternative Prediction Model to Guide Treatment

Additionally, we established an alternative prediction model for therapy success using a machine learning classifier (Figure S3A,B). Two linear support vector machines (SVM) were each trained on the AUCs, with labels “response” and “no response” according to the patients’ treatment outcome. Then the prediction performance was tested in an independent sample (PDO 07). The prediction of the classifier was correct, predicting therapy failure for first-line therapy regimen FOLFIRINOX and tumor response to second-line gemcitabine/nab-paclitaxel in this patient.

Two different approaches of drug response prediction, a simplified prediction score as described above, as well as a machine learning classifier, can aid decision-making for treatment response based on personalized PDO cultures.

### 3.6. PDO Prediction and Potential Clinical Impact

**Figure 4:**
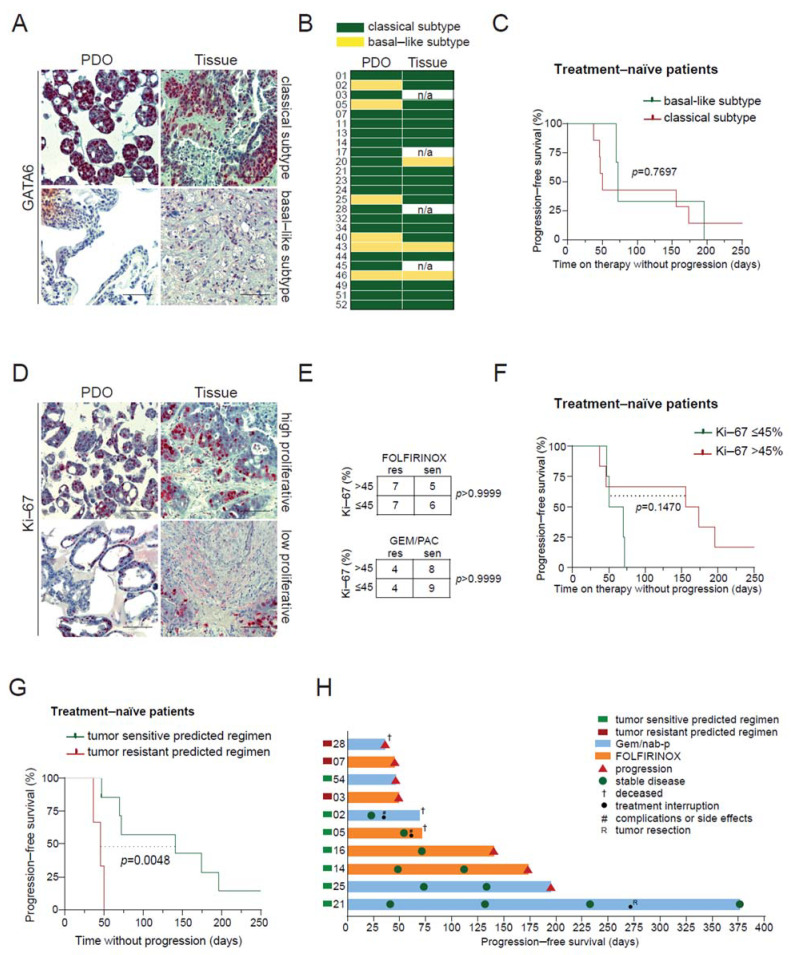
Patient-derived organoid-based drug efficacy prediction and potential clinical impact. (**A**), Immunohistochemistry staining for GATA6 in patient-derived organoids (PDOs) 34 and 43 and corresponding tumor tissue from liver metastases. (**B**), Distribution of classical (green) and basal-like (yellow) subtypes across PDOs and corresponding tumor tissues. (**C**), Kaplan-Meier analysis of progression-free survival (PFS) of treatment-naïve advanced pancreatic cancer (PC) patients according to their classical or basal-like subtype status. (**D**), Immunohistochemistry staining for Ki-67 in PDOs 34 and 49 and corresponding tumor tissue. A tumor cell Ki-67 positivity ≤ 45% shows low proliferation, while Ki-67 >45% denotes high proliferation. (**E**), Contingency table showing proliferation index and sensitivity to chemotherapy regimen in vitro. (**F**), Kaplan-Meier analysis of PFS of treatment-naïve advanced PC patients according to the tumor mitotic index. (**G**) Kaplan-Meier analysis of PFS of treatment-naïve advanced PC patients who received a tumor sensitive or tumor resistant predicted regimen following our PDO-based model. (**H**) Swimmer plot of PFS from treatment-naïve patients. Scale bars represent 100 µm. PDO, patient-derived organoids; FOLFIRINOX, 5-fluorouracil, leucovorin, irinotecan, and oxaliplatin; Gem/nab-p, gemcitabine, and nanoparticle albumin-bound paclitaxel; res, resistant; sen, sensitive.

Several tissue markers have been proposed to have a prognostic impact on PC. As such, GATA6 expression allows a distinction between classical and basal-like subtypes [26]—with the latter being associated with chemoresistance and poor prognosis [27,28,29], while a high Ki-67 proliferation index is considered to have a negative impact on disease-free survival [30,31,32]. All available PDOs with the corresponding tumor tissue were evaluated for GATA6 and Ki-67 expression by immunohistochemistry (Figure 4A,D). The classical subtype was found in 85.7% (18/21) of tissue specimen and in 76.0% (19/25) of PDOs, while the basal-like subtype was encountered less frequently (14.3% (3/21) and 24.0% (6/25), respectively). Concordance between PDOs and corresponding tissue samples was 76.2% (16/21) with a certain drift towards the basal-like subtype upon establishment of PDO cultures [25] (Figure 4B). Of note, a high proliferation index in the PDO did not impact chemosensitivity in vitro (Figure 4E). Stratification of the entire cohort, as well as the treatment-naïve patient cohort according to their molecular subtype in the PDO, did not reveal significant differences in progression-free survival (PFS) (Figure 4C). A trend towards a longer PFS was observed in patients with a high proliferation rate in the PDO, which did not reach statistical significance (*p* = 0.1479) (Figure 4F). To challenge our predictions for the putative clinical value of a PDO-informed treatment in terms of outcome, we compared the PFS of previously treatment-naïve patients with advanced PC who underwent chemotherapy with regimens that were predicted to be either sensitive or resistant. Contrasting with subtype and proliferation, the administration of the tumor sensitive predicted therapy indeed led to a significantly longer patient PFS in the treatment-naïve cohort (mPFS 141 vs. 47 days, *p* = 0.0048) (Figure 4G,H).

### 3.7. Case Report: Integration of Patient-Derived Organoids into Medical Care

**Figure 5:**
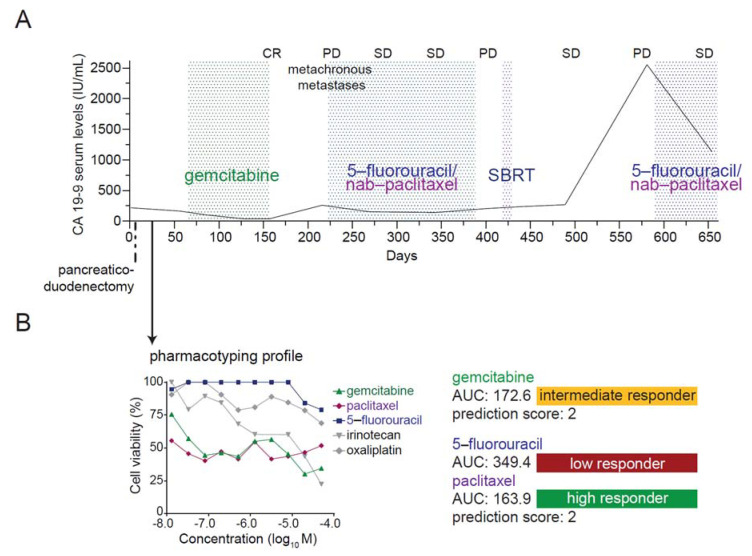
A clinical case demonstrating the feasibility of organoid-tailored therapy. (**A**), Treatment, diagnosis, and response to therapy and CA 19–9 serum levels of the patient. Time intervals for administered treatments are indicated between dotted lines. Patient-derived organoids (PDOs) were isolated from surgical specimens at the time of primary diagnosis of pancreatic ductal adenocarcinoma. The pharmacotyping profile was available after 32 days. (**B**), Cell viability analysis of gemcitabine, paclitaxel, irinotecan, 5-fluorouracil (5-FU), and oxaliplatin treatment in PDO 17 with the corresponding area under the curve (AUC) and classification into low, intermediate, or high responder category. CR, complete response; PD, progressive disease; SBRT, stereotactic body radiation therapy; SD, stable disease; AUC, area under the curve.

A 76-year-old female patient with pancreatic head cancer underwent a pancreaticoduodenectomy with radical lymphadenectomy (pT2, pN1 (3/25), cM0) (Figure 5A). PDOs were established from the surgical specimen, and the pharmacotyping profile was available before initiation of adjuvant chemotherapy with gemcitabine after R1 resection (pharmacotyping available at day 32, treatment start day 64 postsurgery) (Figure 5B). Restaging after three months of adjuvant treatment showed no evidence of disease. Due to putative gemcitabine-related pneumonitis requiring hospitalization, treatment had to be interrupted, and the CT scan after two months of treatment break showed metachronous liver and lymph node metastases concurrent with increasing CA 19–9 values. A second-line palliative treatment with nab-paclitaxel—in combination with 5-fluorouracil for putative synergistic effects and due to intolerance to gemcitabine [33]—was administered, which achieved a long-lasting tumor control in accordance with the PDO pharmacotyping profile. A break in the systemic treatment, due to stereotactic body radiation therapy (SBRT) of a single progressive retroperitoneal lymph, eventually led again to tumor progression, which could be stabilized by re-induction with 5-fluorourcil and nab-paclitaxel. This clinical case shows that PDO-educated decision-making is feasible in first and second-line therapy and may provide clinical benefit.

Pharmacotyping may also identify patients with chemo-refractory disease. PDO 03 was predicted to be a low responder to all tested agents (Figure S4A), which was confirmed as this patient progressed both on first-line therapy with FOLFIRINOX and second-line therapy with gemcitabine/nab-paclitaxel (Figure 3B). Therefore, we explored the feasibility of a second-intent drug screening with further agents that were chosen among substances routinely employed in other cancers. Tested PDOs exhibited heterogeneous responses to these agents (Figure S4B–G), albeit drug testing would have to be conducted on the whole PDO library to allow adequate drug response classification and robust prediction. Thus, extensive drug testing might help to identify active drugs in such cases.

## 4. Discussion

Organoid technology has opened a new era in personalized oncology with broad and promising clinical implications. However, a prospective investigation comparing the pharmacotyping profile with therapeutic response and clinical outcome in a larger number of PC patients is, to our knowledge, not yet available and its potential in clinical routine remains elusive. Therefore, we conducted a prospective evaluation of the feasibility and potential clinical benefit of PDO generation and pharmacotyping across a representative cohort of PC patients.

Several studies have previously shown that PDOs can be isolated from resection or biopsy specimen derived from the primary tumor or metastases at the time of the primary diagnosis [18,20,34,35,36,37]. However, a direct comparison of PDO generation efficacies across studies remains complicated as different definitions of a successful PDO culture establishment exist. The latter refers to either the emergence of organoid structures within the first three passages [18] or the successful propagation beyond passage five [34], whereas a variable proportion of PDOs fails to expand and provide enough biomaterial for further characterization or drug testing. From a clinical perspective, drug response prediction is the paramount goal of developing such an in vitro tool. Therefore, we propose the term pharmacotyping efficacy referring to the proportion of PDOs undergoing preclinical drug testing. Our study showed a tendency towards a higher pharmacotyping efficacy in PDOs established from liver metastases compared to primary tumor biopsies in treatment-naïve patients (78.6% vs. 50%; *p* = 0.1424), highlighting US-guided biopsies of liver metastases as a reliable source for organoid propagation. In primary tumor specimen, the pharmacotyping efficacy relevantly declined in samples obtained by percutaneous biopsies (27.2%, n = 11) compared to EUS-guided FNBs (100%, n = 3) or surgical resections (100%, n = 2). Discrepancies in the establishment success rate between primary tumor specimen and metastases do not seem to be caused by a distinct tumor biology, as several previous studies revealed high genetic similarities between primary and metastatic PC lesions [38,39], but rather by the tissue sampling method. The reduced PDO propagation in primary tumor specimens by percutaneous biopsy might be explained by a potentially lower yield of tumor tissue, due to technical challenges, such as a low tumor burden and the difficult anatomical localization of the pancreas. Seventy-five percent of patients who underwent a biopsy of the primary tumor were classified as a locally advanced or resectable tumor stage, while all patients undergoing liver biopsy suffered from metastatic disease (Table 1). Pharmacotyping efficacy of PDOs from liver metastases, by trend, declined in pretreated patients compared to treatment-naïve patients (64.3% vs. 78.6%; *p* = 0.6776), which might be attributed to a reduced proportion of viable cancer cells, in line with previous studies demonstrating limited engraftment of pretreated patient tumors in mouse models [40]. The time span between the last exposure to chemotherapy and the tissue biopsy did not impact the pharmacotyping efficacy in pretreated patients.

As the efficiency of a combinatorial regimen is driven by the most potent single agent [15], we tested single drugs on our PDO library. Testing single substances offer the advantage to select novel combinations of high-potential drugs, while avoiding ineffective substances and thereby toxicity, which frequently impedes longer-term treatment and severely impacts quality of life in clinical practice. We developed two different approaches for drug response prediction: a machine learning classifier to predict success or failure to FOLFIRINOX or gemcitabine/nab-paclitaxel, as well as a simplified prediction score for daily routine that offers the advantage to select novel combinations. Based on our prediction score, our model accurately predicted therapeutic response in treatment-naïve patients with a very high accuracy of 91.1%. The administration of a treatment anticipated to be efficient translated into a clinical benefit with a significantly longer PFS (mPFS 141 vs. 46 days; *p* = 0.0048). In patients with resectable cancer, adjuvant chemotherapy may be selected organoid-based, as PDOs can be expanded for drug testing within the time frame of postoperative recovery. In the palliative setting, however, there is only a narrow window to begin systemic treatment, and first-line therapy must be initiated before pharmacotyping profiles are obtained. Pharmacotyping profiles were available after a median time of 53 days, in some cases, however, as early as after 21 days. In cases of early prediction of therapy failure, an immediate switch towards a tumor sensitive predicted regimen might be considered without awaiting confirmation of progression in restaging CT. Such a potential approach, however, needs to be evaluated in a randomized setting.

We further challenged our model to predict second-line therapeutic response. Our prediction model was still valid (accuracy of 80.0%), suggesting that treatment-naïve organoids harbor prognostic value to guide both first and second-line therapy. Pharmacotyping may also identify treatment-naïve patients who are likely to have a chemo-refractory disease. In these patients, the performance of an extended drug screening to identify potential effective targeted agents, early initiation of panel-sequencing, or participation in a clinical trial should be considered. The accuracy of response prediction substantially declined in PDOs derived from pretreated patients (accuracy of 40.0%), particularly in those with more than one line of chemotherapeutic pretreatment. In two out of three patients with only one prior chemotherapy line, the prediction was correct. In patients with more than one prior therapy line, our model did not prognosticate therapy efficacy in a reliable manner. This might be explained by chemoresistance mechanisms mediated by components of the tumor microenvironment [41,42] increasingly emerging after several lines of treatment that the organoid model fails to fully recapitulate. PDOs are composed of pure neoplastic populations, whereas a multi-cell type organoid coculture model of the tumor microenvironment [43] might be a more valuable tool to mirror resistance mechanisms in heavily pretreated patients. Additional measures, such as panel- or whole-exome sequencing of the organoids or single-cell RNA-sequencing of the primary specimen, together with an extended drug panel for PDO screening, might help to circumvent this limitation.

The limitations of our study are the limited number of patients who finally qualified for a correlation between the in vitro prediction and the individual patient response. In a proportion of the included patients, a correlation was not possible, as PC was not confirmed in histology, pharmacotyping was unsuccessful, or patients did not receive any chemotherapy or restaging, due to rapid deterioration of performance status or death. To further improve the benefit of such a PDO-based preclinical model, a larger drug panel should be screened to identify subgroups of patients who are susceptible to targeted agents. Up to 26% of the PC profiles harbor actionable molecular alterations, and matched therapies for those patients substantially improved their overall survival [2]. As best-in-class examples, PARP inhibitors targeting germline *BRCA1* or *BRCA2* mutations [44], TRK inhibitors for *NTRK1, NTRK2*, or *NTRK3* fusions [45], and immune checkpoint inhibitors for tumors with DNA mismatch repair (MMR)-deficiency or high microsatellite instability (MSI-H) [46] have been approved in PC. Hence, systematic screening of multiple agents may reveal susceptibilities and improve treatment options in selected subgroups. Novel methods that are currently under investigation, such as automated systems or microfluidic platforms requiring a smaller amount of biomass, may further optimize workflows and facilitate high-throughput screening.

## 5. Conclusions

In summary, we demonstrated that ex vivo pharmacotyping of PDOs is feasible within a reasonable time frame in treatment-naïve and pretreated PC patients regardless of the tumor stage. Pharmacotyping efficacy was best in PDOs isolated from treatment-naïve liver metastases, highlighting it as a reliable source for organoid propagation. We identified a positive correlation between drug sensitivity prediction and therapeutic response in treatment-naïve patients for first and second-line treatment, indicating that PC patients may benefit from a PDO-educated treatment. The potential benefit of pharmacotyping-aided clinical decision-making is underlined by the significantly longer PFS under the administration of a therapy anticipated to be efficient. Importantly, we further conclude that this particular model does not seem to be beneficial for heavily pretreated patients with more than one line of prior chemotherapy, as the power of prediction significantly declined in this setting. This work paves the road toward organoid-based precision medicine, albeit its effectiveness needs to be evaluated in randomized clinical trials.

## Figures and Tables

**Table 1:** Characteristics of pancreatic cancer patients. No, number; ECOG, Eastern Cooperative Oncology Group; US, ultrasound; EUS, endoscopic ultrasound; FNB, fine needle biopsy; PDO, patient-derived organoid; FOLFIRINOX, 5-fluorouracil, leucovorin, irinotecan, and oxaliplatin; OFF, 5-fluorouracil, leucovorin and oxaliplatin; nab-p, nab-paclitaxel; nal-iri/5-FU, nanoliposomal irinotecan, 5-fluorouracil and leucovorin, FOLFOX, 5-fluorouracil, leucovorin, and oxaliplatin; Doce/Ox, Docetaxel, and oxaliplatin; CR, complete response; PR, partial response; PD, progressive disease; SD, stable disease.

	Untreated	Treated
**Patients**, no.	30	14
**Age at diagnosis**, mean (range) in years	66.6 (41–81)	59.0 (44–67)
**Sex**, no. (%)		
Male	18 (60)	9 (64)
Female	12 (40)	5 (36)
**Tumor stage**, no. (%)		
Metastasized	18 (60)	14 (100)
Locally advanced	8 (27)	0 (0)
Resectable	4 (13)	0 (0)
**ECOG**, no. (%)		
0	19 (63)	9 (64)
1	10 (33)	5 (36)
2	1 (3)	0 (0)
**Sampling method**, no. (%)		
US-guided biopsy	25 (83)	14 (100)
EUS-guided FNB	3 (10)	0 (0)
Surgical resection	2 (7)	0 (0)
**Localization of biopsy**, no. (%)		
Primary tumor	16 (53)	0 (0)
Liver metastases	14 (47)	14 (100)
**Pharmacotyping**, no. (%)		
Success	19 (63)	9 (64)
Failure	11 (37)	5 (36)
**Prior systemic therapy lines**, no. (%)		
1	0 (0)	4 (29)
2	0 (0)	6 (43)
≥3	0 (0)	4 (29)
**Prior therapy before PDO generation**, no. (%)		
Platinum-based (FOLFIRINOX, OFF, carboplatin/nab-p)	0 (0)	13 (93)
Gemcitabine/nab-p	0 (0)	11 (79)
Surgical resection	0 (0)	4 (29)
Radio(chemo)therapy	0 (0)	2 (14)
Nal-iri/5-FU	0 (0)	1 (7)
**Treatment regimen after PDO generation**, no. (%)		
Platinum-based (FOLFIRINOX, FOLFOX, Doce/Ox)	12 (40)	1 (7)
Gemcitabine/(nab-p)	11 (37)	2 (14)
No (adjuvant/palliative) chemotherapy	6 (20)	4 (29)
Surgical resection	4 (13)	0 (0)
Nal-iri/5-FU	0 (0)	3 (21)
Clinical trial	0 (0)	3 (21)
Olaparib	0 (0)	1 (7)
**Restaging**, no. (%)		
Response (CR, PR, SD)	13 (43)	4 (29)
No response (PD)	9 (30)	6 (43)
Not available	8 (27)	4 (29)

## Data Availability

The data presented in this study are included in this published article.

## Supplementary Materials

The following are available online at https://www.mdpi.com/article/10.3390/cancers13112539/s1, Figure S1: PDO pharmacotyping is a reliable method, Figure S2: Change of target lesion size upon chemotherapy treatment, Figure S3: Machine learning classifier for prediction of treatment success, Figure S4: Subgrouping of the PDO library, Table S1: Patients’ samples submitted to pharmacotyping.
