# Supplementary material for: A Prospective Feasibility Trial to Challenge Patient–Derived Pancreatic Cancer Organoids in Predicting Treatment Response
Source: Cancers (Basel). 2021 May 21;13(11):2539. doi: 10.3390/cancers13112539 (PMC8196829; doi:10.3390/cancers13112539)
Supplement: Supplementary file 1 [file cancers-13-02539-s001.zip › cancers-1175229-supplementary.pdf]

A

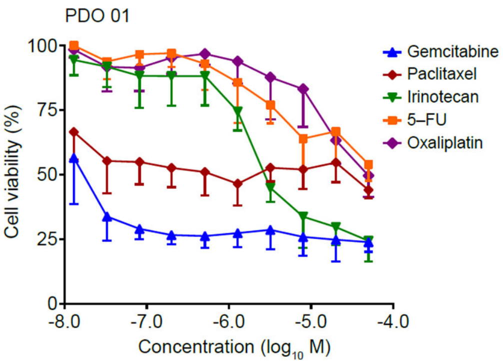

C

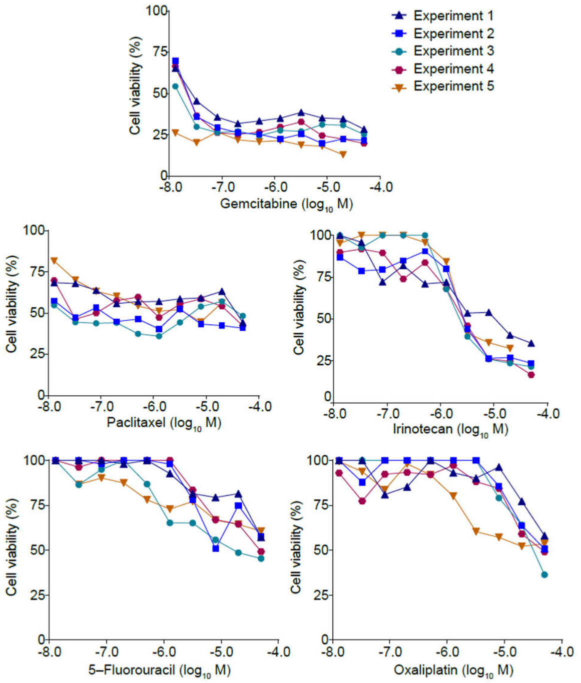

B

	AUC	95% CI
Gemcitabine	104.6	92.0 to 117.2
Paclitaxel	189.2	175.1 to 203.3
Irinotecan	238.5	222.9 to 254.2
5–FU	299.2	283.6 to 314.8
Oxaliplatin	309.7	293.1 to 326.3

D

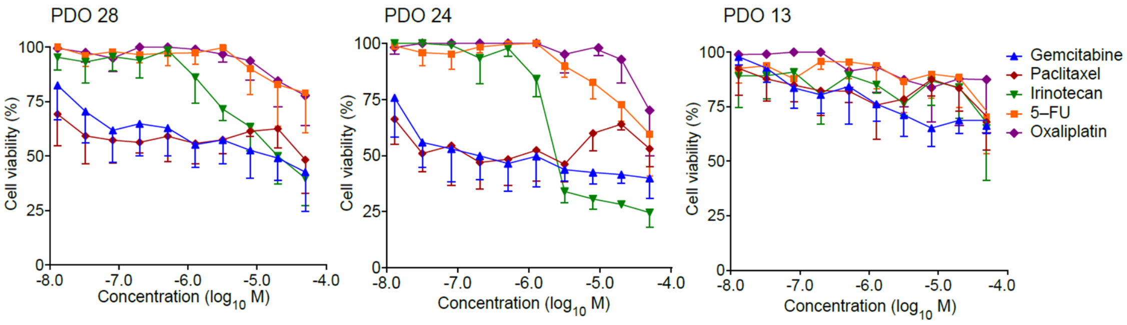

Supplementary Figure S1. PDO pharmacotyping is a reliable method.
(**A**), Cell viability analysis of gemcitabine, paclitaxel, irinotecan, 5–fluorouracil (5–FU), and oxaliplatin treatment in patient-derived organoid (PDO) line 01. Data are represented as mean ± SD of five independent experiments performed by four experimenters. (**B**), Areas under the curves and 95% confidence intervals (CI) of the cell viability analysis shown in **A**. (**C**), Independent cell viability assays of gemcitabine, paclitaxel, irinotecan, 5-FU, and oxaliplatin treatment in patient-derived organoid line 01 used for the viability analysis shown in **A**. (**D**), Cell viability analysis of gemcitabine, paclitaxel, irinotecan, 5–FU, and oxaliplatin treatment in patient-derived organoid lines 28, 24, and 13. Data are represented as mean ± SD of three independent experiments. CI, confidence interval; SD; standard deviation; AUC, area under the curve; PDO, patient-derived organoids.

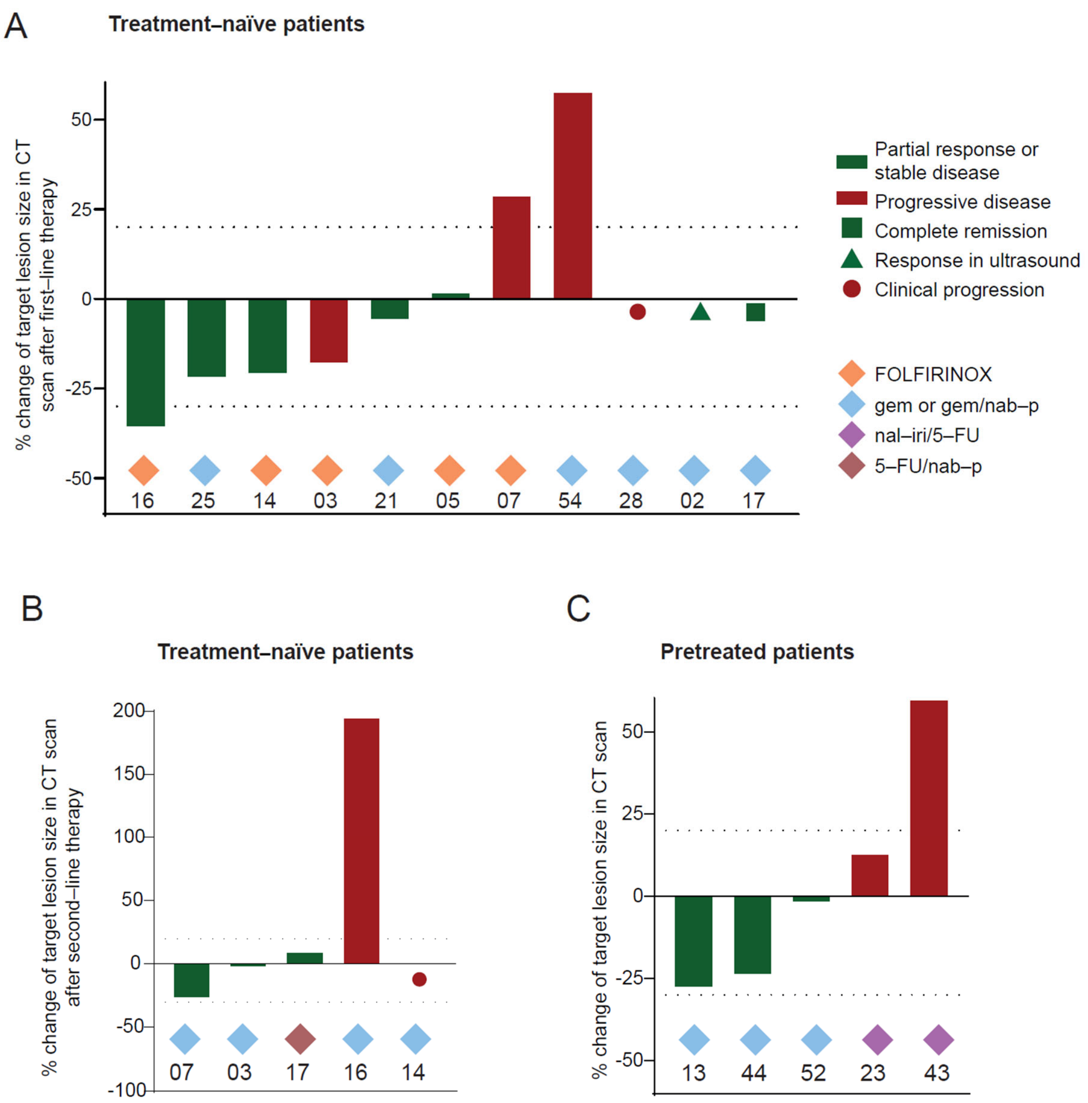

Supplementary Figure S2. Change of target lesion size upon chemotherapy treatment.
Waterfall plot showing percentage change in target lesion size in restaging CT scan based on RECIST 1.1 criteria, (**A**), after first–line treatment and (**B**), after second–line treatment in treatment–naïve patients. (**C**), Waterfall plot showing change in target lesion size in CT scan based on RECIST 1.1 criteria after subsequent treatment in pretreated patients. CT, computed tomography; FOLFIRINOX, 5–fluorouracil, leucovorin, irinotecan, and oxaliplatin; GEM/nab-p, gemcitabine, and nanoparticle albumin–bound paclitaxel; nal-iri/5–FU, nanoliposomal irinotecan, 5–fluorouracil, and leucovorin; 5–FU/nab-p, 5–fluorouracil, leucovorin, and nanoparticle albumin–bound paclitaxel; RECIST, Response Evaluation Criteria In Solid Tumors.

A

Response from FOLFIRINOX-SVM:

$$-5.27 \times AUC_{IRI} - 16.15 \times AUC_{5FU} - 16.38 \times AUC_{OX} + 11519.14 > 0$$

Response from Gem/nab-p-SVM:

$$-9.575 \times AUC_{GEM} + 14.10279 \times AUC_{PAC} - 558.7679 > 0$$

B

PDO	03	05	14	16	07	02	17	21	25	28	54
Therapy	FOLFIRINOX				FOLFIRINOX / Gem/nab-p	Gem/nab-p					
Label	-1	1	1	1	-1 / 1	1	1	1	1	-1	-1
	support vectors	support vectors	support vectors				support vectors			support vectors	support vectors
Prediction FOLFIRINOX-SVM	-1	1	1	1	-1	1	-1	-1	-1	-1	-1
Prediction Gem/nab-p-SVM	1	1	1	1	1	1	1	1	1	-1	-1

= not used for training (blue) = response (green) = no response (red)

Supplementary Figure S3. Machine learning classifier for prediction of treatment success.
(**A**)**,** Formular to determine response from FOLFIRINOX and Gem/nab-p support vector machines (SVM) based on the areas under curves (AUCs) for the single drugs as indicated. (**B**)**,** Two linear SVMs were each trained on the AUCs of untreated 11 PDOs, labeled response (green) and no response (red) according to the patients' treatment outcome. Prediction of FOLFIRINOX and Gem/nab-p–SVM as indicated (response (green), no response (red)). The performance of the classifier was tested on an independent sample (PDO 07), correctly predicting treatment outcome. FOLFIRINOX, 5–fluorouracil, leucovorin, irinotecan, and oxaliplatin; Gem/nab–p, gemcitabine and nanoparticle albumin-bound paclitaxel; AUC, area under the curve; SVM, support vector machine.

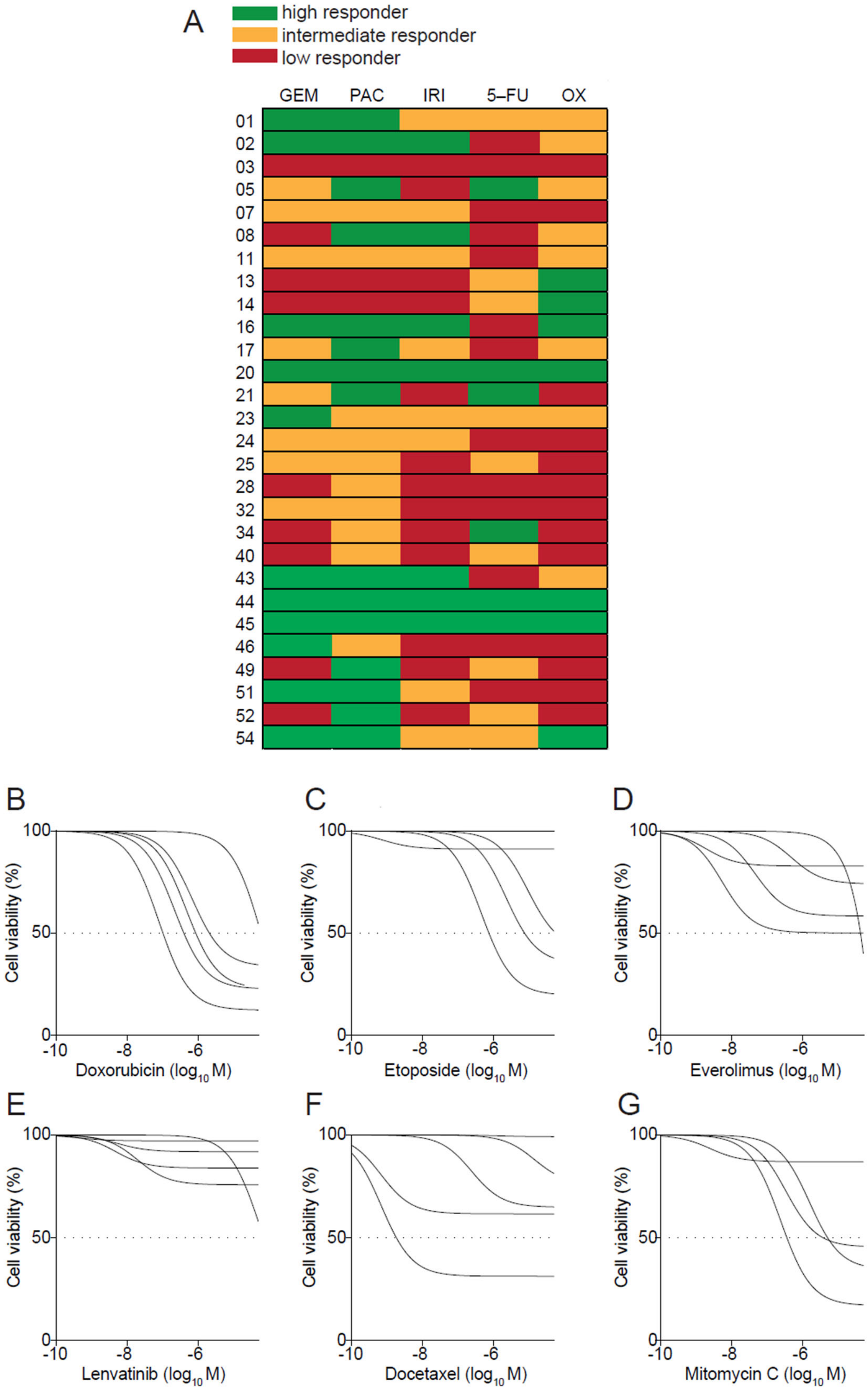

Supplementary Figure S4. **Subgrouping of the PDO library.**

(**A**)**,** Classification into three subgroups (high responder (green), intermediate responder (orange), and low responder (red)) of all tested PDO lines, based on AUC clustering by the Jenks natural breaks

classification method. (**B-G**)**,** Cell viability analysis of (**B**), doxorubicin (**C**), etoposide (**D**), everolimus (**E**), lenvatinib (**F**), docetaxel, and (**G**), mitomycin C treatment in PDOs. 5–FU, 5–fluorouracil; GEM, gemcitabine; IRI, irinotecan; OX, oxaliplatin; PAC, paclitaxel; PDO, patient-derived organoids.

**Patients' samples for pharmacotyping**, no.	54
**Final diagnosis of pancreatic masses**, no. (%)	
Malignant epithelial tumors of the pancreas	
Ductal adenocarcinoma, G1–3	41 (76)
Undifferentiated anaplastic carcinoma, G4	1 (2)
Squamous cell carcinoma	1 (2)
Acinar cell carcinoma, G2	1 (2)
Pancreatic neuroendocrine neoplasms	
Neuroendocrine tumor, G1 or 2	3 (6)
Neuroendocrine carcinoma, G3	1 (2)
Benign tumors	
Serous cystadenoma	1 (2)
Other tumor entities	
T-cell non-hodgkin lymphoma	1 (2)
No diagnostic tissue or necrosis	4 (7)

Supplementary Table S1. Patients' samples submitted to pharmacotyping.
No, number; G, grade.

PDO	Age	Sex	ECOG	Stage	Sampling	Biopsy	Histology	Prior therapy lines	Treatment after PDO generation	Staging	PFS (days)
1	49	male	1	metastasized	US	liver metastasis	ductal adenocarcinoma	2	none	n/a	n/a
2	69	male	2	metastasized	US	liver metastasis	ductal adenocarcinoma	0	Gem/nab–p	SD	70
3	42	male	0	metastasized	US	liver metastasis	ductal adenocarcinoma	0	FOLFIRINOX	PD	50
5	65	male	1	metastasized	US	liver metastasis	ductal adenocarcinoma	0	FOLFIRINOX	SD	72
7	41	male	0	metastasized	US	liver metastasis	ductal adenocarcinoma	0	FOLFIRINOX	PD	46
8	76	female	1	metastasized	US	liver metastasis	ductal adenocarcinoma	0	none	n/a	n/a
11	77	male	1	locally advanced	US	primary tumor	ductal adenocarcinoma, G1	0	Radiotherapy	n/a	n/a
13	67	female	0	metastasized	US	liver metastasis	ductal adenocarcinoma	4	Gem/nab–p	SD	n/r
14	63	male	0	metastasized	US	liver metastasis	ductal adenocarcinoma	0	FOLFIRINOX	SD	174
16	53	female	0	metastasized	US	primary tumor	ductal adenocarcinoma, G3	0	FOLFIRINOX	PR	141
17	76	female	0	resectable	surgery	primary tumor	ductal adenocarcinoma, G2	0	Adj. gemcitabine	CR	156
20	57	female	2	metastasized	US	liver metastasis	ductal adenocarcinoma	0	FOLFIRINOX	n/a	n/a
21	69	female	0	locally advanced	US	primary tumor	ductal adenocarcinoma, G2-3	0	Gem/nab–p	SD	365, n/r
23	58	male	0	metastasized	US	liver metastasis	ductal adenocarcinoma	2	Nal–iri/5–FU	PD	92
24	41	male	1	metastasized	US	liver metastasis	ductal adenocarcinoma	2	Docetaxel/Oxaliplatin	PD	64
25	61	male	0	metastasized	US	liver metastasis	ductal adenocarcinoma	0	Gem/nab–p	SD	196
28	77	female	0	metastasized	EUS	primary tumor	ductal adenocarcinoma	0	Gem/nab–p	PD	37
32	77	female	1	resectable	surgery	primary tumor	ductal adenocarcinoma, G2	0	none	n/a	n/a
34	59	male	1	metastasized	US	liver metastasis	ductal adenocarcinoma	2	none	n/a	n/a
40	57	male	0	resectable	EUS	primary tumor	ductal adenocarcinoma, G2	0	none	n/a	n/a
43	61	male	1	metastasized	US	liver metastasis	ductal adenocarcinoma	1	Nal–iri/5–FU	PD	56
44	45	female	0	metastasized	US	liver metastasis	ductal adenocarcinoma	1	Gem/nab–p	SD	n/r
45	69	male	1	metastasized	US	liver metastasis	ductal adenocarcinoma	0	none	n/a	n/a
46	63	female	0	metastasized	US	liver metastasis	ductal adenocarcinoma	1	Olaparib	n/a	n/a
49	66	female	0	resectable	surgery	primary tumor	ductal adenocarcinoma, G1	0	FOLFIRINOX adjuvant	n/a	n/a
51	81	male	0	metastasized	US	liver metastasis	squamous cell carcinoma	0	FOLFOX	n/a	n/a
52	70	male	0	metastasized	US	liver metastasis	ductal adenocarcinoma	1	Gem/nab–p	SD	n/r
54	64	male	1	metastasized	US	liver metastasis	undifferentiated carcinoma, G4	0	Gem/nab–p	PD	47

Supplementary Table S2. Overview of pharmacotyped patient cohort with regard to baseline characteristics, histology, prior therapy lines, therapy initiated after PDO isolation, staging, and PFS. PDO, patient-derived organoid; PFS, progression–free survival; 5–FU, 5–fluorouracil; EUS, endoscopic ultrasound; US, ultrasound; FOLFIRINOX, 5-fluorouracil, leucovorin, irinotecan, and oxaliplatin; FOLFOX, 5–fluorouracil, leucovorin, and oxaliplatin; GEM, gemcitabine; Nal–iri, nanoliposomal irinotecan; PAC, paclitaxel; CR, complete response; PD, progressive disease; PR, partial response; SD, stable disease; n/a, not available; n/r, not reached.
